# How phenomenology can help us learn from the experiences of others

**DOI:** 10.1007/s40037-019-0509-2

**Published:** 2019-04-05

**Authors:** Brian E. Neubauer, Catherine T. Witkop, Lara Varpio

**Affiliations:** 1 Department of Medicine, Uniformed Services University, Bethesda, MD USA; 2 General Internal Medicine Service, Walter Reed National Military Medical Center, Bethesda, MD USA; 3 Department of Preventative Medicine and Biostatistics, Uniformed Services Medical Center, Bethesda, MD USA

**Keywords:** Transcendental phenomenology, Hermeneutic phenomenology, Qualitative

## Abstract

**Introduction:**

As a research methodology, phenomenology is uniquely positioned to help health professions education (HPE) scholars learn from the experiences of others. Phenomenology is a form of qualitative research that focuses on the study of an individual’s lived experiences within the world. Although it is a powerful approach for inquiry, the nature of this methodology is often intimidating to HPE researchers. This article aims to explain phenomenology by reviewing the key philosophical and methodological differences between two of the major approaches to phenomenology: transcendental and hermeneutic. Understanding the ontological and epistemological assumptions underpinning these approaches is essential for successfully conducting phenomenological research.

**Purpose:**

This review provides an introduction to phenomenology and demonstrates how it can be applied to HPE research. We illustrate the two main sub-types of phenomenology and detail their ontological, epistemological, and methodological differences.

**Conclusions:**

Phenomenology is a powerful research strategy that is well suited for exploring challenging problems in HPE. By building a better understanding of the nature of phenomenology and working to ensure proper alignment between the specific research question and the researcher’s underlying philosophy, we hope to encourage HPE scholars to consider its utility when addressing their research questions.

A Qualitative Space highlights research approaches that push readers and scholars deeper into qualitative methods and methodologies. Contributors to *A Qualitative Space *may: advance new ideas about qualitative methodologies, methods, and/or techniques; debate current and historical trends in qualitative research; craft and share nuanced reflections on how data collection methods should be revised or modified; reflect on the epistemological bases of qualitative research; or argue that some qualitative practices should end. Share your thoughts on Twitter using the hashtag: #aqualspace

## Introduction

*Human beings, who are almost unique in having the ability to learn from the experience of others, are also remarkable for their apparent disinclination to do so.—Douglas Adams*

Despite the fact that humans are one of few animals who can learn from the experiences of others, we are often loath to do so. Perhaps this is because we assume that similar circumstances could never befall us. Perhaps this is because we assume that, if placed in the same situation, we would make wiser decisions. Perhaps it is because we assume the subjective experience of an individual is not as reliably informative as objective data collected from external reality. Regardless of the assumptions grounding this apprehension, it is essential for scholars to learn from the experiences of others. In fact, it is a foundational premise of research. Research involves the detailed study of a subject (i. e., an individual, groups of individuals, societies, or objects) to discover information or to achieve a new understanding of the subject [1]. Such detailed study often requires understanding the experiences of others so that we can glean new insights about a particular phenomenon. Scholars in health professions education (HPE) are savvy to the need to learn from the experiences of others. To maximize the effectiveness of feedback, of workplace-based learning, of clinical reasoning, or of any other of a myriad of phenomena, HPE researchers need to be able to carefully explore and learn from the experiences of others. What often curtails these efforts is a lack of methodology. In other words: HPE researchers need to know *how *to learn from the experiences of others.

Phenomenology is a qualitative research approach that is uniquely positioned to support this inquiry. However, as an approach for engaging in HPE research, phenomenology does not have a strong following. It is easy to see why: To truly understand phenomenology requires developing an appreciation for the philosophies that underpin it. Those philosophies theorize the meaning of human experience. In other words, engaging in phenomenological research requires the scholar to become familiar with the philosophical moorings of our interpretations of human experience. This may be a daunting task, but Douglas Adams never said learning from the experiences of others would be easy.

The questions that phenomenology can answer, and the insights this kind of research can provide, are of foundational importance to HPE: What is the experience of shame and the impact of that experience for medical learners [2]? What does it mean to be an empathetic clinician [3]? What is the medical learner’s experience of failure on high stakes exams [4]? How do experienced clinicians learn to communicate their clinical reasoning in professional practice [5]? Answers to such questions constitute the underpinnings of our field. To answer such questions, we can use phenomenology to learn from the experiences of others.

In this manuscript, we delve into the philosophies and methodologies of two varieties of phenomenology: *hermeneutic *and *transcendental. *Our goal is not to simplify the complexities of phenomenology, nor to argue that all HPE researchers should use phenomenology. Instead, we suggest that phenomenology is a valuable approach to research that needs to have a place in HPE’s body of research. We will place these two approaches in the context of their philosophical roots to illustrate the similarities and differences between these ways of engaging in phenomenological research. In so doing, we hope to encourage HPE researchers to thoughtfully engage in phenomenology when their research questions necessitate this research approach.

## What is phenomenology?

In simple terms, phenomenology can be defined as an approach to research that seeks to describe the essence of a phenomenon by exploring it from the perspective of those who have experienced it [6]. The goal of phenomenology is to describe the meaning of this experience—both in terms of *what *was experienced and *how* it was experienced [6]. There are different kinds of phenomenology, each rooted in different ways of conceiving of the *what *and *how *of human experience. In other words, each approach of phenomenology is rooted in a different school of philosophy. To choose a phenomenological research methodology requires the scholar to reflect on the philosophy they embrace. Given that there are many different philosophies that a scientist can embrace, it is not surprising that there is broad set of phenomenological traditions that a researcher can draw from. In this manuscript, we highlight the transcendental and the hermeneutic approaches to phenomenology, but a broader phenomenological landscape exists. For instance, the Encyclopedia of Phenomenology, published in 1997, features articles on seven different types of phenomenology [7]. More contemporary traditions have also been developed that bridge the transcendental/hermeneutic divide. Several of these traditions are detailed in Tab. 1 [8–10].

**Table 1:** Description of three contemporary approaches to phenomenology

Phenomenological approach	Description	Key figures
Lifeworld research	A blended approach that explores how daily experiences manifest in the lifeworld of individuals through consideration of selfhood, sociality, embodiment, temporality, and spatiality [8]	Peter Ashworth, Karin Dahlberg
Post-intentional phenomenology	A blended approach that treats the phenomenon as the unit of analysis but asserts that phenomena are multiple, partial, contextual, and in flux; being simultaneously produced and producing [9]	Mark Vagle
Interpretive phenomenological analysis (IPA)	A blended approach that aims to provide detailed examination of the lived experience of a phenomenon through participant’s personal experiences and personal perception of objects and events. In contrast to other approaches, in IPA the researcher performs an active role in the interpretive process [10]	Jonathan Smith

To understand any of these approaches to phenomenology, it is useful to remember that most approaches hold a similar definition of phenomenology’s object of study. Phenomenology is commonly described as the study of phenomena as they manifest in our experience, of the way we perceive and understand phenomena, and of the meaning phenomena have in our subjective experience [11]. More simply stated, phenomenology is the study of an individual’s lived experience of the world [12]. By examining an experience as it is subjectively lived, new meanings and appreciations can be developed to inform, or even re-orient, how we understand that experience [13].

From this shared understanding, we now address how *transcendental (descriptive) phenomenology *and* hermeneutic (interpretive) phenomenology *approach this study in different ways. These approaches are summarized in Tab. 2.

**Table 2:** Comparison of transcendental and hermeneutic phenomenology

	Transcendental (descriptive) phenomenology	Hermeneutic (interpretive) phenomenology
Philosophical origins	Husserl	Heidegger Gadamer
Ontological assumptions	Reality is internal to the knower; what appears in their consciousness	Lived experience is an interpretive process situated in an individual’s lifeworld
Epistemological assumptions	Observer must separate him/herself from the world including his/her own physical being to reach the state of the transcendental I; bias-free; understands phenomena by descriptive means	Observer is part of the world and not bias free; understands phenomenon by interpretive means
Researcher role in data collection	Bracket researcher subjectivity during data collection and analysis	Reflects on essential themes of participant experience with the phenomenon while simultaneously reflection on own experience
Researcher role in data analysis/writing	Consider phenomena from different perspectives, identify units of meaning and cluster into themes to form textural description (the what of the phenomenon). Use imaginative variation to create structural (the how) description. Combine these descriptions to form the essence of the phenomenon	Iterative cycles of capturing and writing reflections towards a robust and nuanced analysis; consider how the data (or parts) contributed to evolving understanding of the phenomena (whole)
Methodological texts	Polkinghorne [28] Moustakas [18] Giorgi [27]	Van Manen [12]
Examples	Takavol [32]	Bynum [2]

## Transcendental phenomenology

Phenomenology originates in philosophical traditions that evolved over centuries; however, most historians credit Edmund Husserl for defining phenomenology in the early 20th century [14]. Understanding some of Husserl’s academic history can provide insight into his transcendental approach to phenomenology. Husserl’s initial work focused on mathematics as the object of study [15], but then moved to examine other phenomena. Husserl’s approach to philosophy sought to equally value both objective and subjective experiences, with his body of work ‘culminating in his interest in “pure phenomenology” or working to find a universal foundation of philosophy and science [13].’ Husserl rejected positivism’s absolute focus on objective observations of external reality, and instead argued that phenomena *as perceived by the individual’s consciousness *should be the object of scientific study. Thus, Husserl contended that no assumptions should inform phenomenology’s inquiry; no philosophical or scientific theory, no deductive logic procedures, and no other empirical science or psychological speculations should inform the inquiry. Instead, the focus should be on what is given directly to an individual’s intuition [16]. As Staiti recently argued, this attitude towards phenomenology is akin to that of ‘a natural scientist who has just discovered a previously unknown dimension of reality [17].’ This shift in focus requires the researcher to return ‘to the self to discover the nature and meaning of things [18].’ As Husserl asserted: ‘Ultimately, all genuine and, in particular, all scientific knowledge, rests on inner evidence [19].’ Inner evidence—that is, what appears in consciousness—is where a phenomenon is to be studied. What this means for Husserl is that subjective and objective knowledge are intimately intertwined. To understand the reality of a phenomenon is to understand the phenomenon as it is lived by a person. This lived experience is, for Husserl, a dimension of *being *that had yet to be discovered [17]. For Husserl, phenomenology was rooted in an epistemological attitude; for him, the critical question of a phenomenological investigation was ‘What is it for an individual to know or to be conscious of a phenomenon [20]?’ In Husserl’s conception of phenomenology, any experienced phenomenon could be the object of study thereby pushing analysis beyond mere sensory perception (i. e. what I see, hear, touch) to experiences of thought, memory, imagination, or emotion [21].

Husserl contended that a lived experience of a phenomenon had features that were commonly perceived by individuals who had experienced the phenomenon. These commonly perceived features—or universal essences—can be identified to develop a generalizable description. The essences of a phenomenon, according to Husserl, represented the true nature of that phenomenon. The challenge facing the researcher engaging in Husserl’s phenomenology, then, is:

*To describe things in themselves, to permit what is before one to enter consciousness and be understood in its meanings and essences in the light of intuition and self-reflection. The process involves a blending of what is really present with what is imagined as present from the vantage point of possible meanings; thus, a unity of the real and the ideal *[18]*.*

In other words, the challenge is to engage in the study of a person’s lived experience of a phenomenon that highlights the universal essences of that phenomenon [22]. This requires the researcher to suspend his/her own attitudes, beliefs, and suppositions in order to focus on the participants’ experience of the phenomenon and identify the essences of the phenomenon. One of Husserl’s great contributions to philosophy and science is the method he developed that enables researchers ‘to suspend the natural attitude as well as the naïve understanding of what we call the human mind and to disclose the realm of transcendental subjectivity as a new field of inquiry [17].’

In Husserl’s’ transcendental phenomenology (also sometimes referred to as the *descriptive* approach), the researcher’s goal is to achieve *transcendental subjectivity*—a state wherein ‘the impact of the researcher on the inquiry is constantly assessed and biases and preconceptions neutralized, so that they do not influence the object of study [22].’ The researcher is to stand apart, and not allow his/her subjectivity to inform the descriptions offered by the participants. This lived dimension of experience is best approached by the researcher who can achieve the state of the *transcendental I*—a state wherein the objective researcher moves from the participants’ descriptions of facts of the lived experience, to universal essences of the phenomenon at which point consciousness itself could be grasped [23]. In the state of the *transcendental I*, the researcher is able to access the participants’ experience of the phenomenon pre-reflectively—that is ‘without resorting to categorization on conceptualization, and quite often includes what is taken for granted or those things that are common sense [13].’ The *transcendental I *brings no definitions, expectations, assumption or hypotheses to the study; instead, in this state, the researcher assumes the position of a *tabula rasa, *a blank slate, that uses participants’ experiences to develop an understanding of the essence of a phenomenon.

This state is achieved via a series of reductions. The first reduction, referred to as the *transcendental stage*, requires transcendence from the natural attitude of everyday life through *epoche*, also called the process of *bracketing*. This is the process through which the researchers set aside—or bracket off as one would in a mathematical equation—previous understandings, past knowledge, and assumptions about the phenomenon of interest. The previous understandings that must be set aside include a wide range of sources including: scientific theories, knowledge, or explanation; truth or falsity of claims made by participants; and personal views and experiences of the researcher [24]. In the second phase, *transcendental-phenomenological reduction*, each participant’s experience is considered individually and a complete description of the phenomenon’s meanings and essences is constructed [18]. Next is reduction via *imaginative variation *wherein all the participants’ descriptions of conscious experience are distilled to a unified synthesis of essences through the process of free variation [25]. This process relies on intuition and requires imagining multiple variations of the phenomenon in order to arrive at the essences of the phenomenon [25]. These essences become the foundation for all knowledge about the phenomenon.

The specific processes followed to realize these reductions vary across researchers engaging in transcendental phenomenology. One commonly used transcendental phenomenological method is that of psychologist Clark Moustakas, and other approaches include the works of: Colaizzi [26], Giorgi [27], and Polkinghorne [28]. Regardless of the approach used, to engage rigorously in transcendental phenomenology, the researcher must be vigilant in his/her bracketing work so that the researcher’s individual subjectivity does not bias data analysis and interpretations. This is the challenge of reaching the state of the *transcendental I *where the researcher’s own interpretations, perceptions, categories, etc. do not influence the processes of reduction. It is important to note that modern philosophers continue to wrestle with Husserl’s notions of bracketing. If bracketing is successfully achieved, the researcher sets aside the world and the entirety of its content—including the researcher’s physical body [17]. While dedication to this bracketing is challenging to maintain, Husserl asserts that it is necessary. Suspending reliance on and foundations in physical reality is the only way to abandon our human experiences in such a way as to find the *transcendent I. *Researchers might borrow [29] practices from other qualitative research methods to achieve this goal. For instance, a study could be designed to have multiple researchers triangulate [30] their reductions to confirm appropriate bracketing was maintained. Alternatively, a study could involve validation of data [18] via member checking [31] to ensure that the identified essences resonated with the participants’ experiences.

Husserl’s transcendental phenomenology has been employed by HPE researchers. For example, in 2012, Tavakol et al. studied medical students’ understanding of empathy by engaging in transcendental phenomenological research [32]. The authors note that medial students’ loss of empathy as they transition from pre-clinical to clinical training is well documented in the medical literature [33], and has been found to negatively impact patients and the quality of healthcare provided [34]. Tavakol et al. [32] used a descriptive phenomenological approach (i. e. using the methodology of Colaizzi and Giorgi) to report on the phenomenon of empathy as experienced by medical students during the course of their training. The authors identified two key factors impacting empathic ability: innate capacity for empathy and barriers to displaying empathy [32].

## Hermeneutic phenomenology

Hermeneutic phenomenology, also known as interpretive phenomenology, originates from the work of Martin Heidegger. Heidegger began his career in theology, but then moved into academia as a student of philosophy. While Heidegger’s philosophical inquiry began in alignment with Husserl’s work, he later challenged several key aspects of Husserl’s transcendental phenomenology. A foundational break from his predecessor was the focus of phenomenological inquiry. While Husserl was interested in the nature of knowledge (i. e., an epistemological focus), Heidegger was interested in the nature of being and temporality (i. e., an ontological focus) [21]. With this focus on human experience and how it is lived, hermeneutic phenomenology moves away from Husserl’s focus on ‘acts of attending, perceiving, recalling and thinking about the world [13]’ and on human beings as knowers of phenomenon. In contrast, Heidegger is interested in human beings as actors in the world and so focuses on the relationship between an individual and his/her *lifeworld. *Heidegger’s term *lifeworld *referred to the idea that ‘individuals’ realities are invariably influenced by the world in which they live [22].’ Given this orientation, individuals are understood as *always already *having an understanding of themselves within the world, even if they are not constantly, explicitly and/or consciously aware of that understanding [17]. For Heidegger, an individual’s conscious experience of a phenomenon is not separate from the world, nor from the individual’s personal history. Consciousness is, instead, a formation of historically lived experiences including a person’s individual history and the culture in which he/she was raised [22]. An individual cannot step out of his/her *lifeworld. *Humans cannot experience a phenomenon without referring back to his/her background understandings. Hermeneutic phenomenology, then, seeks ‘to understand the deeper layers of human experience that lay obscured beneath surface awareness and how the individual’s *lifeworld, *or the world as he or she pre-reflectively experiences it, influences this experience [35].’ Hermeneutic phenomenology studies individuals’ narratives to understand what those individuals experience in their daily lives, in their *lifeworlds.*

But the hermeneutic tradition pushes beyond a descriptive understanding. Hermeneutic phenomenology is rooted in interpretation—interpreting experiences and phenomena via the individual’s *lifeworld. *Here, Heidegger’s background in theology can be seen as influencing his approach to phenomenology. Hermeneutics refers to the interpretation of texts, to theories developed from the need to translate literature from different languages and where access to the original text (e. g., the Bible) was problematic [36]. If all human experience is informed by the individual’s *lifeworld, *and if all experiences must be interpreted through that background, hermeneutic phenomenology must go beyond description of the phenomenon, to the interpretation of the phenomenon. The researcher must be aware of the influence of the individual’s background and account for the influences they exert on the individual’s experience of being.

This is not to say that the individual’s subjective experience—which is inextricably linked with social, cultural, and political contexts—is pre-determined. Heidegger argued that individuals have *situated freedom. Situated freedom *is a concept that asserts that ‘individuals are free to make choices, but their freedom is not absolute; it is circumscribed by the specific conditions of their daily lives [22].’ Hermeneutic phenomenology studies the meanings of an individual’s being in the world, as their experience is interpreted through his/her *lifeworld, *and how these meanings and interpretations influence the choices that the individual makes [13]. This focus requires the hermeneutic phenomenologist to interpret the narratives provided by research participants in relation to their individual contexts in order to illuminate the fundamental structures of participants’ understanding of being and how that shaped the decisions made by the individual [37].

Another key aspect that distinguishes hermeneutic phenomenology is the role of the researcher in the inquiry. Instead of bracketing off the researcher’s subjective perspective, hermeneutic phenomenology recognizes that the researcher, like the research subject, cannot be rid of his/her *lifeworld. *Instead, the researcher’s past experiences and knowledge are valuable guides to the inquiry. It is the researcher’s education and knowledge base that lead him/her to consider a phenomenon or experience worthy of investigation. To ask the research to take an unbiased approach to the data is inconsistent with hermeneutic phenomenology’s philosophical roots. Instead, researchers working from this tradition should openly acknowledge their preconceptions, and reflect on how their subjectivity is part of the analysis process [16].

The interpretive work of hermeneutic phenomenology is not bound to a single set of rule-bound analytical techniques; instead, it is an interpretive process involving the interplay of multiple analysis activities [35]. In general, this process:

*Starts with identifying an interesting phenomenon that directs our attention towards lived experience. Members of the research team then investigate experience as it is lived, rather than as it is conceptualized, and reflect on the essential [phenomenological] themes that characterize the participant’s experience with the phenomenon, simultaneously reflecting on their own experiences. Researchers capture their reflections in writing and then reflect and write again, creating continuous, iterative cycles to develop increasingly robust and nuanced analyses. Throughout the analysis, researchers must maintain a strong orientation to the phenomenon under study (i.* *e., avoid distractions) and attend to the interactions between the parts and the whole. This last step, also described as the hermeneutic circle, emphasizes the practice of deliberately considering how the data (the parts) contribute to the evolving understanding of the phenomena (the whole) and how each enhances the meaning of the other *[35]*.*

In the hermeneutic approach to phenomenology, theories can help to focus inquiry, to make decisions about research participants, and the way research questions can be addressed [22]. Theories can also be used to help understand the findings of the study. One scholar whose engagement with hermeneutic phenomenology is widely respected is Max van Manen [38]. Van Manen acknowledges that hermeneutic phenomenology ‘does not let itself be deceptively reduced to a methodical schema or an interpretative set of procedures [39].’ Instead, this kind of phenomenology requires the researcher to read deeply into the philosophies of this tradition to grasp the project of hermeneutic phenomenological thinking, reading, and writing.

A recent study published by Bynum et al. illustrates how hermeneutic phenomenology may be employed in HPE [2]. In this paper, Bynum et al. explored the phenomenon of shame as an emotion experienced by medical residents and offer insights into the effects of shame experiences on learners. As a means in scholarly inquiry, this study demonstrates how hermeneutic phenomenology can provide insight into complex phenomena that are inextricably entwined in HPE.

## Conclusion

Incorporating phenomenological research methodologies into HPE scholarship creates opportunities to learn from the experiences of others. Phenomenological research can broaden our understanding of the complex phenomena involved in learning, behaviour, and communication that are germane to our field. But success in these efforts is dependent upon both improved awareness of the potential value of these approaches, and enhanced familiarization with the underlying philosophical orientation and methodological approaches of phenomenology. Perhaps most critically, HPE scholars must construct research processes that align with the tenets of the methodology chosen and the philosophical roots that underlie it. This alignment is the cornerstone for establishing research rigour and trustworthiness.

Following a specific checklist of verification activities or mandatory processes cannot buoy the quality and rigour of a particular phenomenological study. Instead, beyond maintaining fidelity between research question, paradigm, and selected methodology, robust phenomenological research involves deep engagement with the data via reading, reflective writing, re-reading and re-writing. In Moustakas’s approach to transcendental phenomenology, the researcher reads the data, reduces the data to meaning units, re-reads those reductions to then engage in thematic clustering, compares the data, writes descriptions, and so on in an ongoing process of continually engaging with the data and writing reflections and summaries until the researcher can describe the essence of the lived experience [18]. In hermeneutic phenomenology, scholars describe engaging in a hermeneutic circle wherein the researcher reads the data, constructs a vague understanding, engages in reflective writing, then re-engages with the text with revised understandings [40]. In cycles of reading and writing, of attending to the whole of the text and the parts, the hermeneutic researcher constructs an understanding of the lived experience. In both traditions, deep engagement with the data via reading, writing, re-reading and re-writing is foundational. While this engagement work is not standardized, Polkinghorne suggests that rich descriptions of phenomenological research might be characterized by qualities such as vividness, richness, accuracy, and elegance [41]. While we question how these qualities might be evaluated in a qualitative study, they confirm that attention to the depth of engagement in reading and writing of the phenomenological data is a necessary condition for rigour.

Phenomenology is a valuable tool and research strategy. For those who are not familiar with its philosophical underpinnings or methodological application, it can seem challenging to apply to HPE scholarship. We hope this manuscript will serve to relieve some of the apprehension in considering the use of phenomenology in future work. We believe that the appropriate application of phenomenology to HPE’s research questions will help us to advance our understanding by learning from the experiences of others.

### Disclaimer

The views expressed herein are those of the authors and do not necessarily reflect those of the Uniformed Services University of the Health Sciences, the United States Department of Defense or other federal agencies.
